# Care network types and depression among people with cognitive impairment: A latent class analysis

**DOI:** 10.1371/journal.pone.0357737

**Published:** 2026-09-10

**Authors:** Mina Hwang, Yeji Hwang

**Affiliations:** 1 College of Nursing, Seoul National University, Seoul, Republic of Korea; 2 Center for World-leading Human-care Nurse Leaders for the Future by Brain Korea 21 (BK 21) four project, Seoul National University, Seoul, Republic of Korea; 3 Research Institute of Nursing Science, Seoul National University, Seoul, Republic of Korea; University of Bamberg, GERMANY

## Abstract

Depression is a common neuropsychiatric symptom in people with cognitive impairment. Although care networks, defined as groups of caregivers supporting an individual with cognitive impairment, may influence depression, evidence regarding this relationship remains limited. The purpose of this study was to identify the care network types among people with cognitive impairment and assess their association with depression. Using a nationally representative sample of older adults, 537 people with cognitive impairment who required assistance with activities of daily living were analyzed. Latent class analysis was employed to identify care network types according to the composition of six categories of care providers: spouses, children, extended family members, distant relatives/friends/neighbors, privately paid caregivers, and public caregivers. Multiple linear regression was then used to examine the associations between care network types and depression after adjusting for covariates. Five care network types were identified: Child-centered (*n* = 175, 32.6%), Spouse-centered (*n* = 101, 18.8%), Friends and Neighbors-centered (*n* = 43, 8.0%), Public-centered (*n* = 97, 18.1%), and Mixed (*n* = 121, 22.5%). Compared with the Child-centered Care Network, the Public-centered Care Network (*β* = 0.09, *p* = 0.041) and the Mixed Care Network (*β* = 0.14, *p* = 0.002) were associated with higher depression scores. In pairwise comparisons, the Public-centered and Mixed Care Networks also had significantly higher depression scores than the Child-centered, Spouse-centered, and Friends and Neighbors-centered Care Networks. These findings suggest that the overall configuration of caregivers may be relevant to depression among people with cognitive impairment and highlight the importance of considering care network composition when assessing their psychological well-being.

## Introduction

Depression is a common neuropsychiatric symptom among people with cognitive impairments such as dementia or mild cognitive impairment [1]. The prevalence of depression is approximately 39% among people with cognitive impairment [1]. Because depression can lead to faster cognitive decline, greater caregiver burden, and accelerated nursing home placement [2–4], it is essential to understand factors associated with depression. Depression among people with cognitive impairment might not only be determined by neurodegenerative changes or individual factors, but also by caregiver- and environment-related factors [5–7]. This view is consistent with the conceptual model of behavioral and psychological symptoms of dementia proposed by Kales et al. [6]. As people with cognitive impairment experience progressive loss of multiple cognitive abilities and functional decline [8], they require support from caregivers regarding activities of daily living [9]. Owing to the extensive amount of care required, multiple caregivers are often involved in providing care to an individual with cognitive impairment [10–12]. Moreover, changes in family structure and the expansion of public care services have diversified the composition of caregiving arrangements among older adults, resulting in multiple caregivers [13,14].

Care networks, which are defined as the configuration of caregivers surrounding an individual [15], may be associated with psychological well-being. According to the Social Convoy Model, individuals are embedded in dynamic networks of social relationships across the life course, and the structure, function, and quality of these networks are associated with psychological well-being [16]. In this context, care networks surrounding people with cognitive impairment represent networks of social relationships organized around caregiving. This perspective gives rise to the question as to whether caregiver composition is associated with psychological well-being among people with cognitive impairment. However, much of the existing literature has only focused on the primary caregivers, investigating how the relationship of the primary caregiver to the person being cared for, i.e., spouse, child, or others, is associated with psychological outcomes [17–19]. This single-caregiver view might not be adequate for the complexity of modern caregiving.

Given these limitations, a network perspective is needed [14,20,21]. Research conducted in Western countries such as the United States and the Netherlands has identified care network types using latent class analysis based on nationally representative samples of older adults [14,21]. Although these studies have provided valuable insights by identifying diverse care network types, there is a lack of information on care networks in Eastern cultures, where cultural norms place a strong emphasis on expecting adult children to support their parents [14,21], and few studies have focused specifically on older adults with cognitive impairment [13,22]. Additionally, empirical evidence on how the structural composition of care networks relates to psychological well-being among people with cognitive impairment remains scarce. Therefore, the purpose of this study was to identify care network types among people with cognitive impairment through latent class analysis and to examine how these network types are associated with depression.

### Conceptual framework of the study

This study was guided by two conceptual models [6,16]. First, the Social Convoy Model conceptualizes individuals as being embedded in dynamic networks of social relationships across the life course [16]. These networks are characterized by their structure, function, and quality, which are shaped by personal and situational factors and are associated with health and well-being [16]. In line with this model, the present study focuses on the structural composition of caregivers surrounding people with cognitive impairment. Second, Kales et al. [6] proposed a conceptual model for understanding behavioral and psychological symptoms of dementia, such as anxiety and depression, emphasizing the interactions among people with dementia, caregivers, and the environment. In this model, neurodegeneration alters the ability of people with dementia to engage with their caregivers and the environment, and caregiver and environmental factors may contribute to neuropsychiatric symptoms of dementia either independently or in interaction with cognitive impairment [6].

**Fig 1:**
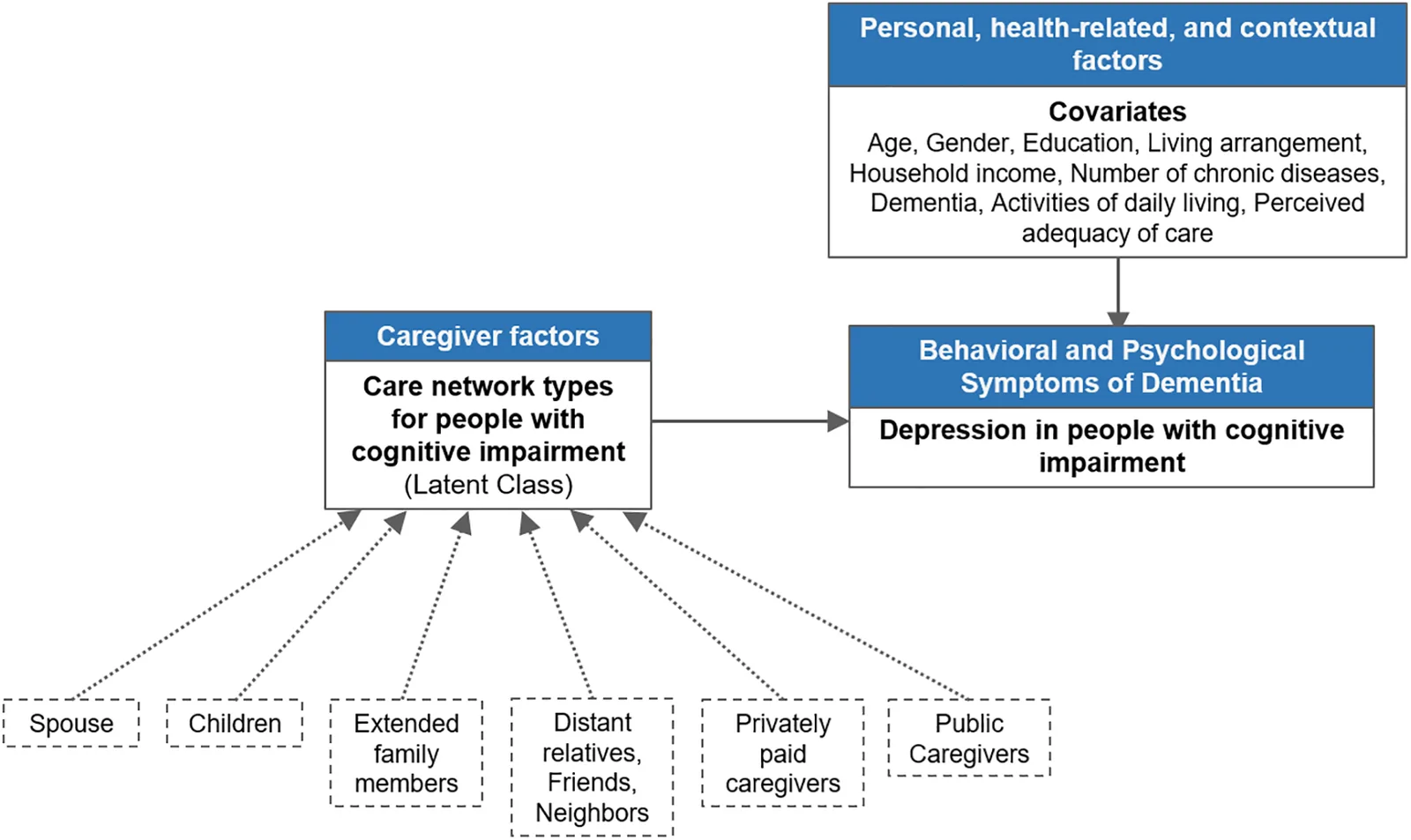
Conceptual framework of the study.

These two models were integrated into the research framework for the study presented here (see Fig 1). The Social Convoy Model [16] provided the basis for conceptualizing care networks in terms of caregiver composition, whereas the model proposed by Kales et al. [6] provided the broader dementia-care context for understanding depression as associated with caregiver- and environment-related factors. Together, these models form the conceptual basis for examining whether different care network types are associated with depression among people with cognitive impairment.

## Materials and methods

### Study design

A cross-sectional, descriptive design was applied in this study based on secondary data from the 2023 National Survey of Older Koreans, a nationally representative survey of community-dwelling older adults aged 65 years or older in South Korea. The Ministry of Health and Welfare and the Korea Institute for Health and Social Affairs conducted the survey to generate foundational data and key indicators for policies related to the health and welfare of older adults [23]. In this survey, stratified sampling was employed to collect representative data from older Korean adults. Data were collected from September to November 2023 via face-to-face interviews conducted by trained interviewers. Interviewers read each survey question slowly and clearly to participants and recorded their responses. The original survey was based primarily on direct responses from older adults. All participants included in this study provided direct responses. Participants provided written informed consent for the original survey.

### Participants

**Fig 2:**
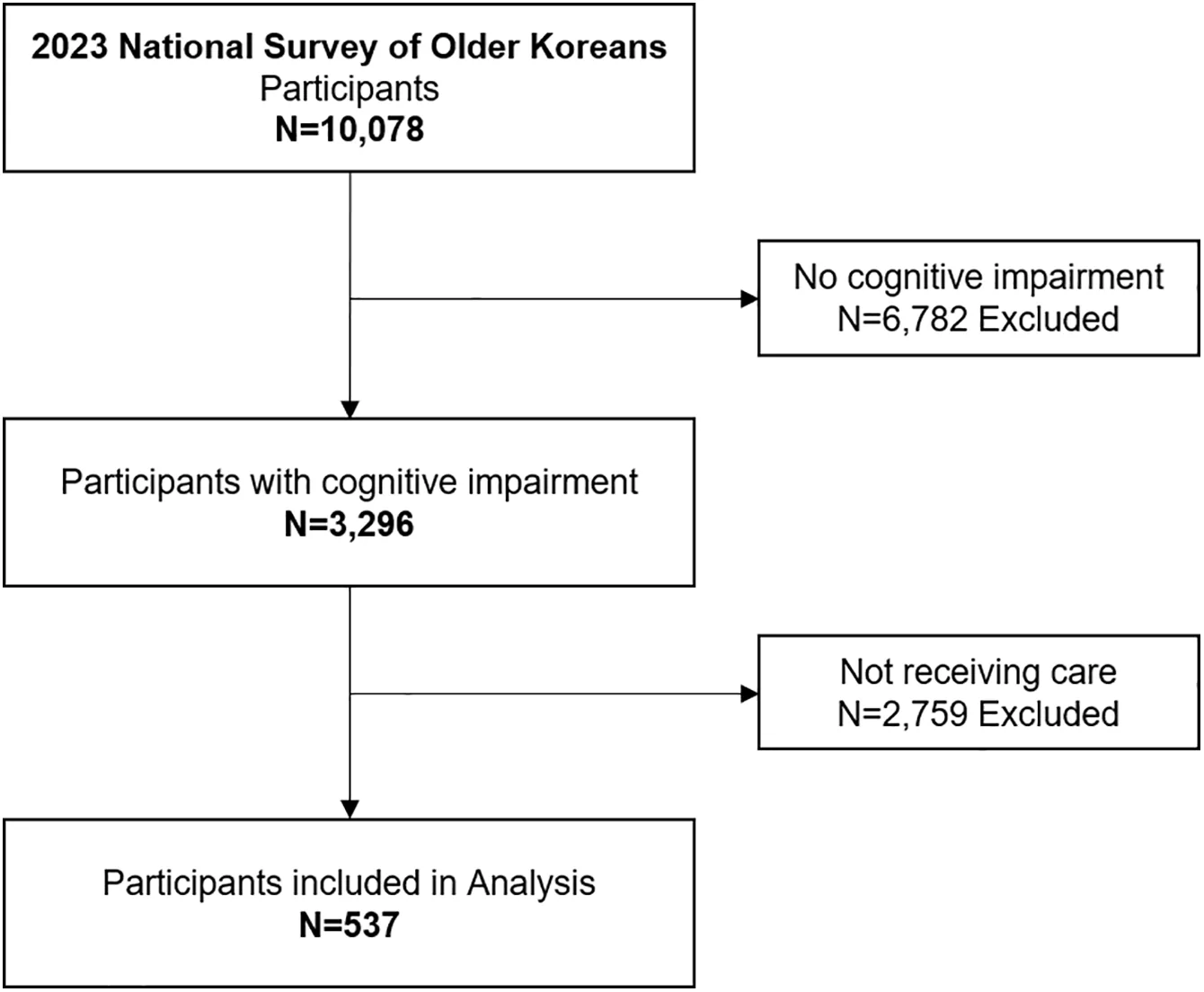
Flow chart of extracting study samples.

As the analysis was conducted to identify care network types among people with cognitive impairment, the analytic sample was limited to older adults with cognitive impairment who received assistance with activities of daily living from caregivers. Cognitive function was assessed in the 2023 National Survey of Older Koreans using the Korean Mini-Mental State Examination, 2nd Edition, Standard Version (K-MMSE-2:SV) [23,24]. The K-MMSE-2:SV yields a raw score ranging from 0 to 30. Participants with raw scores below 24 were classified as having cognitive impairment [24]. Among the original survey population of 10,078 individuals, 537 were included in the present study (Fig 2). This study was approved as an exempt by the Institutional Review Board at the authors’ institution.

### Measures

In this study, three sets of variables were used: (1) depression as the final outcome variable, (2) basic information for determining care network types, and (3) additional variables included as covariates in the regression analyses examining the association between care network types and depression. The measures are described below in this order.

#### Depression.

Depression was measured using the Korean version of the Short Form Geriatric Depression Scale [25]. The scale consists of 15 items addressing feelings and moods over the past week, with responses recorded as ‘yes’ (1) or ‘no’ (0). The total scores range from 0 to 15, with higher scores indicating greater depression [26].

#### Basic information for determining care network types.

In this study, care networks were classified using latent class analysis based on the types of care providers who provided assistance to people with cognitive impairment in performing activities of daily living. The 2023 National Survey of Older Koreans contained questions as to whether the participants received assistance with activities of daily living from others and from whom they received assistance. In the original survey, seven categories of caregivers were distinguished: (1) co-residing family members, (2) non-co-residing family members, (3) relatives, friends, or neighbors, (4) privately paid caregivers, (5) long-term care insurance services, (6) customized public care services for older adults, and (7) other public care services. For respondents who reported receiving assistance from co-residing family members, non-co-residing family members, or relatives, friends, or neighbors, additional questions were used to identify the specific relationship with the caregiver.

Based on the responses, care providers were reclassified into six mutually exclusive groups: (1) spouse; (2) children; (3) extended family members; (4) distant relatives, friends, or neighbors; (5) privately paid caregivers; and (6) public caregivers. In South Korea, public caregivers are available through the Long-Term Care Insurance system and the Customized Care Services for Older Adults; these public caregivers provide care to older adults who need assistance with activities of daily living. The six classification applied here is based on previous research [14,17,21]. Six binary variables were generated for each respondent to indicate the presence (1) or absence (0) of each care provider type; these variables were used as indicators in the latent class analysis.

The spouse category included respondents who received care from their spouse. The child category included adult sons or daughters and their spouses. The extended family members category consisted of grandchildren and their spouses; parents, grandparents, siblings, and their spouses; and in-laws, such as the spouse’s parents, grandparents, or siblings. The distant relatives, friends, or neighbors category included other blood relatives, non-relatives living together, friends, neighbors, and informal social service providers. The category of distant relatives, friends, or neighbors was defined to distinguish less closely related or non-kin social ties from immediate and extended family members, reflecting differences in relational proximity. The privately paid caregiver category referred to privately hired caregivers. The public caregiver category included long-term care insurance services, customized care services for older adults, and other public care services.

#### Covariates.

To control for potential confounding effects when examining the association between care network types and depression, additional variables were included as covariates in the regression analysis. These variables were age, gender, education, living arrangements, annual household income, number of chronic diseases, MMSE score, activities of daily living, and perceived adequacy of care because they are known to be associated with depression in older adults [2,27,28].

Living arrangements were categorized as living alone, living only with a spouse, living with children, or living with others. Annual income level was divided into quintiles, as provided in the original survey, where the first and fifth quintiles represent the poorest and richest groups, respectively. Raw MMSE scores were used to describe the cognitive characteristics of the study participants in Table 1. For the regression analyses, MMSE scores were categorized into three equal-width intervals across the study-specific score range of 0–23 to account for the potential nonlinear association between cognitive function and depression: 0–7, 8–15, and 16–23. Activities of daily living were evaluated using seven items: dressing, washing face and hands, bathing, eating, transfer, toileting, and continence. Each item was scored as fully independent (1), partially dependent (2), or fully dependent (3). The total scores range from 7 to 21, with higher total scores indicating more functional limitations. The perceived adequacy of care was assessed by asking the participants about the sufficiency of care they received in daily life, which was rated as very insufficient (1), insufficient (2), moderate (3), sufficient (4), or very sufficient (5). Higher scores indicated greater perceived adequacy of care.

**Table 1:** Characteristics of the participants (*N* = 537).

Characteristics	Mean (SD) or *n* (%)	Min–Max
**Age**	82.1 (6.45)	65–103
**Gender**		
Male	140 (26.1)	
Female	397 (73.9)	
**Education**		
No education	262 (48.8)	
Elementary school	169 (31.5)	
Middle school	59 (11.0)	
High school	37 (6.9)	
College or above	10 (1.9)	
**Living arrangement**		
Alone	265 (49.3)	
With spouse only	166 (30.9)	
With children	100 (18.6)	
With others	6 (1.1)	
**Annual household income quintile**		
1st (poorest)	195 (36.3)	
2nd	149 (27.7)	
3rd	93 (17.3)	
4th	61 (11.4)	
5th (richest)	39 (7.3)	
**Number of chronic diseases**	3.61 (1.99)	0–15
**Raw MMSE score**	17.50 (4.60)	0–23
**MMSE score category**		
16–23	381 (70.9)	
8–15	142 (26.4)	
0–7	14 (2.6)	
**Activities of daily living score**	8.65 (2.34)	7–21
**Perceived adequacy of care**	3.25 (0.88)	1–5
**Caregiver types (received care from)**		
Spouse	127 (23.6)	
Child	271 (50.5)	
Extended family members	10 (1.9)	
Distant relatives, friends or neighbors	161 (30.0)	
Privately paid caregivers	57 (10.6)	
Public caregivers	244 (45.4)	
**Depression**	6.62 (3.90)	0–15

Note: Participants could receive care from multiple caregiver types.

### Data analyses

Data analyses were performed using the R software (version 4.4.3). Descriptive statistics were used to describe the participants’ characteristics and depression levels. To identify the care network types based on the six care provider groups, latent class analysis was conducted. Latent class analysis is a person-centered statistical approach that identifies unobserved subgroups within a population based on patterns of responses to observed variables, maximizing heterogeneity between groups, while ensuring homogeneity within groups [29]. To determine the optimal number of latent classes (i.e., care network types), we estimated a series of models by sequentially increasing the number of classes from two to six. For each model, the Akaike Information Criterion (AIC), Bayesian Information Criterion (BIC), Bootstrapped Likelihood Ratio Test (BLRT), entropy, and the smallest class size were computed. These measures were used to evaluate the latent class solutions. Lower AIC and BIC values indicate more parsimonious and better fitting models. Entropy reflects clearness of classification. A value of 1 means that each feature is assigned with a probability of 1 to one class and with a probability of 0 to all other classes. A value of 0 means that all features are assigned with the same probability to each class. A value larger than 0.8 is considered acceptable. The recommended smallest class size is at least 5% of the total sample [29,30]. The BLRT was used to assess whether adding an additional class significantly improved model fit, with a *p*-value < 0.05 indicating a significant improvement compared with the model with one fewer class [31]. The number of classes was increased sequentially, and additional classes were retained only if the BLRT indicated a statistically significant improvement in model fit. Entropy and class size were additionally considered to evaluate the quality of classification and the interpretability of the solution.

Chi-square and Kruskal-Wallis H tests, followed by Dunn’s post-hoc tests, were conducted to examine differences in demographic characteristics, health status, and perceived care quality across care network types. Finally, a multiple linear regression analysis was conducted to investigate the relationship between care network type and depression among people with cognitive impairment. Model assumptions, including normality of residuals and homoscedasticity, were assessed. Multicollinearity among the independent variables was examined using generalized variance inflation factors (GVIFs), which indicate how much uncertainty in the estimated effects of a predictor is increased because of its relationships with the other predictors. GVIFs can be used for categorical predictors with multiple levels. A GVIF of 1 indicates no variance inflation. To facilitate comparisons across predictors with different degrees of freedom, GVIF^1/(2×Df)^ values were also reported as adjusted GVIFs [32]. In the first step, only the covariates were entered into the model. In the second step, care network type was added. The additional contribution of care network type in Model 2 was evaluated using a robust Wald test based on the HC3 covariance estimator. HC3 is a heteroscedasticity-consistent covariance matrix estimator [33]. It was used throughout the regression analyses to obtain heteroscedasticity-robust standard errors and associated test statistics. To further examine differences between care network types, pairwise comparisons of adjusted estimated marginal means were conducted for all pairs of care network types based on the regression model.

### Ethical considerations

This study received exempt approval from the Seoul National University Institutional Review Board (IRB No. E2505/004–015). The requirement for informed consent was waived because the study used a de-identified secondary dataset. The authors accessed the data for research purposes on May 19, 2025. Throughout the study, the authors had no access to information that could identify individual participants, as the dataset was fully de-identified.

## Results

The mean age of the study participants was 82.1 (6.45) years (min–max: 65–103 years), and most were female (73.9%). The mean depression score was 6.62 (3.90) (see Table 1).

Latent class analysis was conducted to identify care network types. The model fit statistics for two- to six-class solutions are shown in Table 2. The BLRT was statistically significant for the two- to five-class models, indicating that each model provided a significantly better fit than the model with one fewer class. However, the BLRT for the six-class model was not statistically significant (*p* = 0.060), suggesting that adding a sixth class did not significantly improve model fit compared with the five-class model. The five-class model showed acceptable classification quality, with an entropy value of 0.85, and its smallest class accounted for 8.0% of the sample. Therefore, the 5-class model was selected as the final model.

**Table 2:** Fit statistics for different latent class models.

Class	AIC	BIC	BLRT	Entropy	Number of Parameters	Class distribution rate (%)					
						1	2	3	4	5	6
**2**	3028.34	3084.06	<0.001	0.92	13	20.3	79.7				
**3**	2909.68	2995.40	<0.001	0.88	20	16.8	26.4	56.8			
**4**	2893.70	3009.43	<0.001	0.85	27	32.2	31.3	18.6	17.9		
**5**	**2886.60**	**3032.32**	**<0.001**	**0.85**	**34**	**32.6**	**18.8**	**8.0**	**18.1**	**22.5**	
**6**	2894.57	3070.30	0.060	0.94	41	20.5	16.9	32.0	20.7	8.4	1.5

Note: **Bold** values indicate the selected model. AIC = Akaike Information Criterion. BIC = Bayesian Information Criterion. Lower AIC and BIC values indicate better model fit. BLRT = Bootstrapped Likelihood Ratio Test. Entropy values near 1 ideal and above 0.8 acceptable. The smallest class size should be ≥ 5% for reliable classification.

Item-response probabilities for each of the five latent care network types are shown in Table 3. Item-response probabilities represent the estimated probability that individuals belonging to each latent class receive care from each caregiver type. Class 1 was the largest group, accounting for 32.6% of the sample (*n* = 175). This class was characterized by a probability of 1.00 for receiving care from children, with limited support from public caregivers (0.17). This class was labeled as the “Child-centered Care Network.” Class 2 accounted for 18.8% of participants (*n* = 101). This class was characterized by dominant care from spouses (1.00), with limited involvement from other providers, such as children (0.19). This class was labeled as the “Spouse-centered Care Network.” Class 3 was the smallest group, accounting for 8.0% of the sample (*n* = 43). This class showed the highest probability of receiving care from distant relatives, friends, or neighbors (0.63), with limited support from public caregivers (0.14) and extended family members (0.12). This class was labeled as the “Friends and Neighbors–centered Care Network.” Class 4 accounted for 18.1% of the sample (*n* = 97). This class showed a very high probability of receiving care from public caregivers (0.98), with limited support from privately paid caregivers (0.17). This class was labeled as the “Public-centered Care Network.” Class 5 was the second-largest group, comprising 22.5% of the sample (*n* = 121). This class was characterized by high probabilities of receiving care from public caregivers (0.86), distant relatives, friends, or neighbors (0.84), and children (0.62), along with moderate use of privately paid caregivers (0.26). Given its diverse support structure, this pattern was labeled as the “Mixed Care Network.”

**Table 3:** Item-response probabilities across latent care network classes.

Caregiver	Class 1 (*n* = 175, 32.6%)	Class 2 (*n* = 101, 18.8%)	Class 3 (*n* = 43, 8.0%)	Class 4 (*n* = 97, 18.1%)	Class 5 (*n* = 121, 22.5%)
**Spouse**	0.00	**1.00**	0.02	0.09	0.12
**Children**	**1.00**	0.19	0.00	0.00	**0.62**
**Extended family members**	0.02	0.00	0.12	0.01	0.01
**Distant relatives, Friends, Neighbors**	0.08	0.00	**0.63**	0.00	**0.84**
**Privately paid caregivers**	0.02	0.01	0.00	0.17	**0.26**
**Public caregivers**	0.17	0.02	0.14	**0.98**	**0.86**

Note: **Bold** values indicate caregiver types with relatively high item-response probabilities within each latent class. Item-response probabilities represent the estimated probability of receiving care from each caregiver type within each latent class.

The demographic characteristics, health status, and perceived quality of care of the participants according to care network type are shown in Table 4. Significant differences across the five care network groups were observed for age (p < 0.001), gender (*p* < 0.001), education (*p* < 0.001), living arrangements (*p* < 0.001), annual household income (*p* < 0.001), number of chronic diseases (*p* = 0.009), ADL score (*p* < 0.001), perceived adequacy of care (*p* = 0.004), and depression (*p* < 0.001).

**Table 4:** Characteristics of the study participants by type of care network.

Variables	Child- centered Care (a)	Spouse- centered Care (b)	Friends and Neighbors-centered Care (c)	Public-centered Care (d)	Mixed Care (e)	*p*	Post-hoc (Dunn test)
	n = 175	n = 101	n = 43	*n* = 97	*n* = 121		
	32.6%	18.8%	8.0%	18.1%	22.5%		
	Mean (SD) or *n* (%)	Mean (SD) or *n* (%)	Mean (SD) or *n* (%)	Mean (SD) or *n* (%)	Mean (SD) or *n* (%)		
**Age**	83.06 (5.65)	78.02 (6.08)	79.77 (6.41)	82.70 (6.43)	84.47 (6.18)	<0.001	a, d > b; e > b, c
**Gender**						<0.001	
Male	24 (13.7)	59 (58.4)	13 (30.2)	19 (19.6)	25 (20.7)		
Female	151 (86.3)	42 (41.6)	30 (69.8)	78 (80.4)	96 (79.3)		
**Education**						<0.001	
No education	90 (51.4)	28 (27.7)	24 (55.8)	53 (54.6)	67 (55.4)		
Elementary school	54 (30.9)	33 (32.7)	14 (32.6)	32 (33.0)	36 (29.8)		
Middle school	17 (9.7)	22 (21.8)	2 (4.7)	9 (9.3)	9 (7.4)		
High school	12 (6.9)	12 (11.9)	3 (7.0)	1 (1.0)	9 (7.4)		
College or above	2 (1.1)	6 (5.9)	0 (0.0)	2 (2.1)	0 (0.0)		
**Living arrangement**						<0.001	
Alone	96 (54.9)	0 (0.0)	29 (67.4)	72 (74.2)	68 (56.2)		
With spouse only	19 (10.9)	90 (89.1)	6 (14.0)	21 (21.6)	30 (24.8)		
With children	58 (33.1)	11 (10.9)	5 (11.6)	3 (3.1)	23 (19.0)		
With others	2 (1.1)	0 (0.0)	3 (7.0)	1 (1.0)	0 (0.0)		
**Annual household income quintile**						<0.001	
1st (poorest)	60 (34.3)	18 (17.8)	19 (44.2)	58 (59.8)	40 (33.1)		
2nd	49 (28.0)	26 (25.7)	12 (27.9)	25 (25.8)	37 (30.6)		
3rd	21 (12.0)	35 (34.7)	6 (14.0)	9 (9.3)	22 (18.2)		
4th	25 (14.3)	13 (12.9)	5 (11.6)	2 (2.1)	16 (13.2)		
5th (richest)	20 (11.4)	9 (8.9)	1 (2.3)	3 (3.1)	6 (5.0)		
**Number of chronic diseases**	3.70 (2.03)	3.06 (1.92)	3.63 (2.20)	3.71 (1.73)	3.85 (2.04)	0.009	d, e > b
**MMSE score category**						0.099	
16–23	123 (70.3)	84 (83.2)	29 (67.4)	66 (68.0)	79 (65.3)		
8–15	46 (26.3)	16 (15.8)	12 (27.9)	30 (30.9)	38 (31.4)		
0–7	6 (3.4)	1 (1.0)	2 (4.7)	1 (1.0)	4 (3.3)		
**Activities of daily living score** **(Range: 7–21)**	8.22 (2.06)	8.79 (2.73)	7.53 (1.08)	8.54 (2.07)	9.66 (2.54)	<0.001	e > a, b, c, d; b, d > c
**Perceived adequacy of care** **(Range: 1–5)**	3.34 (0.83)	3.44 (0.99)	2.88 (1.10)	3.23 (0.78)	3.10 (0.79)	0.004	b > c
**Depression** **(Range: 0–15)**	5.87 (3.43)	5.95 (4.21)	6.14 (4.14)	7.33 (3.92)	7.86 (3.83)	<0.001	d > a; e > a, b

Finally, we conducted a multiple linear regression analysis to examine the association between care network type and depression, controlling for demographic characteristics, health status, and adequacy of care (see Table 5). The Child-centered Care Network was used as the reference group as it represented the largest proportion of the sample and reflected the most typical caregiving arrangements in South Korea, where children are generally the primary caregivers for older adults [23,34]. Model 1 included only covariates, whereas Model 2 additionally included care network type. The robust Wald test based on the HC3 estimator indicated that the addition of care network type was statistically significant after adjustment for covariates, F = 4.93, p = 0.001. GVIF values ranged from 1.08 to 3.59, with corresponding aGVIF values of 1.04–1.24, suggesting limited variance inflation among the predictors.

**Table 5:** Multiple linear regression models on depression by care network.

Variables	Model 1					Model 2				
	B	SE	*β*	*p*	GVIF (aGVIF)	B	SE	*β*	*p*	GVIF (aGVIF)
**Care network**										2.52 (1.12)
Child-centered Care						Ref				
Spouse-centered Care						−0.39	0.57	−0.04	0.490	
Friends and Neighbors-centered Care						−0.71	0.61	−0.05	0.246	
Public-centered Care						**0.90**	**0.44**	**0.09**	**0.041**	
Mixed Care						**1.33**	**0.43**	**0.14**	**0.002**	
**Covariates**										
Age	**−0.11**	**0.03**	**−0.18**	**<0.001**	1.25 (1.12)	**−0.13**	**0.03**	**−0.22**	**<0.001**	1.36 (1.16)
Gender					1.27 (1.13)					1.33 (1.15)
Male	Ref					Ref				
Female	**−0.83**	**0.38**	**−0.09**	**0.029**		**−1.01**	**0.39**	**−0.11**	**0.009**	
Education					1.63 (1.06)					1.69 (1.07)
No education	Ref					Ref				
Elementary school	**−0.76**	**0.38**	**−0.09**	**0.047**		**−0.76**	**0.38**	**−0.09**	**0.047**	
Middle school	−0.64	0.58	−0.05	0.270		−0.68	0.58	−0.05	0.243	
High school	**−1.72**	**0.68**	**−0.11**	**0.011**		**−1.76**	**0.66**	**−0.11**	**0.008**	
College or above	−0.44	1.32	−0.02	0.741		−0.32	1.35	−0.01	0.814	
Living arrangement					2.49 (1.16)					3.59 (1.24)
Alone	Ref					Ref				
With spouse only	−0.25	0.39	−0.03	0.532		0.05	0.42	0.01	0.902	
With children	0.33	0.51	0.03	0.523		0.61	0.50	0.06	0.224	
With others only	2.15	1.48	0.06	0.147		2.81	1.50	0.08	0.063	
Annual household income quintile					2.20 (1.10)					2.33 (1.11)
1st (poorest)	Ref					Ref				
2nd	−0.64	0.38	−0.07	0.088		−0.69	0.37	−0.08	0.065	
3rd	−0.93	0.48	−0.09	0.054		**−0.96**	**0.49**	**−0.09**	**0.048**	
4th	−1.07	0.63	−0.09	0.093		−1.17	0.62	−0.10	0.061	
5th (richest)	**−1.73**	**0.78**	**−0.12**	**0.027**		**−1.72**	**0.77**	**−0.11**	**0.026**	
Number of chronic diseases	**0.43**	**0.07**	**0.22**	**<0.001**	1.08 (1.04)	**0.42**	**0.07**	**0.21**	**<0.001**	1.08 (1.04)
MMSE score category					1.30 (1.07)					1.32 (1.07)
16–23	Ref					Ref				
8–15	0.16	0.39	0.02	0.680		0.16	0.39	0.02	0.686	
0–7	0.12	1.18	0.00	0.920		0.38	1.19	0.02	0.750	
Activities of daily living score	**0.43**	**0.07**	**0.26**	**<0.001**	1.11 (1.06)	**0.37**	**0.07**	**0.22**	**<0.001**	1.21 (1.10)
Perceived adequacy of care	**−0.92**	**0.19**	**−0.21**	**<0.001**	1.16 (1.08)	**−0.94**	**0.18**	**−0.21**	**<0.001**	1.18 (1.09)
Constant	14.85	2.39		<0.001		17.14	2.49		<0.001	
R²					0.255					0.282
Adjusted R²					0.229					0.252
*Δ* R²										0.027
*Δ* Wald F(*p*)						4.93 (*p* = .001)				

Note: **Bold** values indicate statistical significance (p < 0.05). GVIF = generalized variance inflation factor, aGVIF = GVIF^1/(2×Df)^, where Df denotes the degrees of freedom associated with the corresponding predictor. The Wald F statistic refers to the HC3 robust Wald test for the addition of care network type in Model 2.

In Model 2, compared with the Child-centered Care Network, participants in the Public-centered Care Network (*β* = 0.09, *p* = 0.041) and the Mixed Care Network (*β* = 0.14, *p* = 0.002) had significantly higher depression scores. No significant differences were observed for the spouse-centered or the friends and neighbors-centered care networks. Among the covariates, age was associated with lower depression (*β* = −0.22, *p* < 0.001). Compared with men, women had lower depression scores (*β* = −0.11, *p* = 0.009). Regarding education, participants with elementary school education (*β* = −0.09, *p* = 0.047) and high school education (*β* = −0.11, *p* = 0.008) had lower depression scores than those with no education. Regarding socioeconomic status, participants in the third income quintile (*β* = −0.09, *p* = 0.048) and fifth income quintile (*β* = −0.11, *p* = 0.026) had significantly lower depression scores compared with those in the lowest quintile. Among health-related variables, the number of chronic diseases (*β* = 0.21, *p* < 0.001) and activities of daily living score (*β* = 0.22, *p* < 0.001) were associated with higher depression, whereas perceived adequacy of care was associated with lower depression (*β* = −0.21, *p* < 0.001).

As shown in Model 2 of Table 5, the adjusted coefficients suggested the following order of depression scores, from lowest to highest: Friends and Neighbors-centered Care Network, Spouse-centered, Child-centered, Public-centered, and Mixed Care Networks. Pairwise comparisons were then conducted based on this adjusted order (see Table 6). Adjusted pairwise comparisons of estimated marginal means showed no significant differences among the Friends and Neighbors-centered, Spouse-centered, and Child-centered Care Networks. Participants in the Public-centered Care Network had significantly higher depression scores than those in the Friends and Neighbors-centered (*p* = 0.015), Spouse-centered (*p* = 0.030), and Child-centered (*p* = 0.041) Care Networks. Similarly, participants in the Mixed Care Network had significantly higher depression scores than those in the Friends and Neighbors-centered (*p* = 0.002), Spouse-centered (*p* = 0.003), and Child-centered (*p* = 0.002) Care Networks. No significant difference was observed between the Public-centered and Mixed Care Networks.

**Table 6:** Adjusted pairwise comparisons of care network types for depression.

Comparison	Mean difference	95% CI	*p*
Friends and Neighbors-centered – Spouse-centered	−0.32	−1.78, 1.15	0.671
Friends and Neighbors-centered – Child-centered	−0.71	−1.92, 0.49	0.246
Friends and Neighbors-centered – Public-centered	**−1.61**	**−2.91, −0.31**	**0.015**
Friends and Neighbors-centered – Mixed	**−2.04**	**−3.36, −0.73**	**0.002**
Spouse-centered – Child-centered	−0.39	−1.52, 0.73	0.490
Spouse-centered – Public-centered	**−1.29**	**−2.46, −0.13**	**0.030**
Spouse-centered – Mixed	**−1.73**	**−2.85, −0.60**	**0.003**
Child-centered – Public-centered	**−0.90**	**−1.76, −0.04**	**0.041**
Child-centered – Mixed	**−1.33**	**−2.18, −0.48**	**0.002**
Public-centered – Mixed	−0.43	−1.42, 0.55	0.387

Note: **Bold** values indicate statistical significance (p < 0.05). Mean differences were calculated as the first group minus the second group. CI = confidence interval.

## Discussion

Using latent class analysis, this study identified five distinct care network groups among people with cognitive impairment: Child-centered, Spouse-centered, Friends and Neighbors–centered, Public-centered, and Mixed Care Networks. These groups reflect the diversity of caregiving structures among people with cognitive impairment, ranging from those centered on a single care provider to those supported by multiple care providers. In the analysis of the association between care network type and depression, older adults in the Public-centered Care Network and Mixed Care Network had significantly higher depression scores than those in the Child-centered Care Network, after adjustment for covariates. Consistent with these findings, in the adjusted pairwise comparisons, older adults in both the Public-centered and Mixed Care Networks had significantly higher depression scores than those in the Child-centered, Spouse-centered, and Friends and Neighbors-centered Care Networks. These findings suggest that variations in care network configuration are associated with depression in people with cognitive impairment.

A closer examination of these five care network types reveals notable differences in their size and composition. The Child-centered Care Network was the most prevalent, followed by the Mixed, Spouse-centered, Public-centered, Friends and Neighbors-centered Care Networks. This pattern contrasts with findings from Western settings, where mixed or publicly supported networks tend to be more common (e.g., [14,21]). The predominance of the Child-centered Care Network in our sample may reflect long-standing family caregiving norms in South Korea [23,34], as well as the tendency for children to become more involved in caregiving as functional dependence increases with cognitive impairment [20,35,36]. The Mixed Care Network, which emerged as the second largest group, also showed substantial involvement of children, further underscoring the central role of intergenerational care in the Korean context.

The Mixed Care Network included care from children but was also characterized by the involvement of multiple other care providers, including public caregivers, distant relatives, friends, neighbors, and privately paid caregivers. These features align with prior evidence that people with cognitive impairment, who typically exhibit higher levels of care dependency, tend to receive support from a larger and more diverse set of caregivers [12,20,35,37]. The relatively older age, higher number of chronic diseases, greater functional limitations, and low perceived care adequacy in this group may indicate particularly high care needs. The combined use of public and privately paid services may also reflect the complementary role of formal care when family resources are limited [38,39].

In the Public-centered Care Network, care was provided predominantly by public caregivers, with little involvement of children, other family members, or friends and neighbors. Participants in this group also had the highest proportion of living alone and the highest proportion in the lowest household income quintile. Similar characteristics have been reported for public or formal care–oriented networks in previous studies [21,40]. These characteristics suggest that public care services may play a particularly important role when informal caregiving resources are limited.

This study examined the association between the care network type and depression. Compared with participants in the Child-centered Care Network, those in the Public-centered and Mixed Care Networks had significantly higher depression scores after adjustment for sociodemographic characteristics, health-related factors, functional limitations, and perceived care adequacy. These findings suggest that care network composition may be one factor associated with depression among older adults with cognitive impairment. This interpretation is consistent with the Social Convoy Model, which emphasizes that the structure and composition of social relationships are linked to psychological well-being [16]. In this sense, the present findings suggest the importance of considering the overall configuration of caregivers, rather than focusing only on the primary caregiver, when examining depression among older adults with cognitive impairment.

Previous findings on the relationship between mixed or diverse care networks and depression have been inconsistent among the general older adult population. Some studies have reported that larger and more diverse networks are associated with higher depression, whereas others have found protective effects of diverse networks [17,41].

One possible interpretation of the association between the Mixed Care Network and higher depression scores is that the involvement of multiple care providers may be a response to greater and more complex care needs rather than an indication of greater support alone. According to the Hierarchical Compensatory Model [38], older adults generally rely first on close family members and turn to more distant kin, non-kin, or formal care providers when preferred family-based care is unavailable or insufficient. In this study, the Mixed Care Network combined care from children with care from distant relatives, friends, neighbors, public caregivers, and privately paid caregivers, and participants in this network had the greatest functional limitations. This pattern may indicate that mixed care arrangements develop in the context of greater care needs or limitations in primary family caregiving resources. Therefore, the higher depression scores observed in this group should be interpreted in relation to both the structure of the care network and the underlying vulnerabilities that may have contributed to the formation of mixed care arrangements.

A second possible explanation concerns coordination and continuity of care when multiple caregivers are involved. The conceptual model of neuropsychiatric symptoms in dementia emphasizes that cognitive impairment, caregiver factors, and environmental factors interact in shaping symptoms such as depression [6]. From this perspective, changes in care arrangements or inconsistencies in care practices may be particularly relevant for people with cognitive impairment, because neurodegenerative changes can alter their ability to engage with caregivers and the surrounding environment. The involvement of multiple caregivers in the Mixed Care Network may increase the need for coordination among caregivers and make maintaining continuity of care more challenging [10,11,42]. Such coordination difficulties have been proposed as one possible explanation for the association between larger care networks and poorer psychological well-being among care recipients [10]. Although these processes were not directly examined in the present study, they may be one possible explanation for the higher depression scores observed among participants in the Mixed Care Network.

The higher depression scores observed in the Public-centered Care Network are consistent with previous findings on formal care networks [19]. One factor that may be relevant to this association is the limited involvement of close informal caregivers. From a task-specific perspective, formal and informal sources of care are not fully interchangeable: formal services are better suited to certain instrumental or technically specialized tasks, whereas informal caregivers such as family members, friends, and neighbors may provide forms of emotional and social support that are less readily replaced by formal services [43]. Previous research also found that older adults in formal care networks had greater loneliness than those in partner or informal care networks, and that loneliness partly explained differences in depressive symptoms between care network types [19]. In this study, the Public-centered Care Network was characterized by predominant involvement of public caregivers and little involvement of children, other family members, or friends and neighbors. Thus, the higher depression scores observed in this group may be related to a care network configuration in which public care is the primary source of care while involvement of close informal caregivers is limited.

### Implications for policy and practice

This study has several implications for the support of people with cognitive impairment and their families. First, it may be beneficial for community nurses to assess not only the primary caregiver but also the broader care network surrounding people with cognitive impairments [44]. Such assessments could include the composition of care providers, perceived adequacy of care, the availability of family and non-family support, and potential needs for care coordination. Given that higher depression scores were observed among participants in the Public-centered and Mixed Care Networks, closer assessment of psychological well-being and care-related needs may be particularly important when public services constitute the main source of care or when multiple care providers are involved. During care transitions, such as the introduction of public services or changes in the primary caregiver, regular monitoring and supportive follow-up may be warranted.

Second, when multiple caregivers are involved, attention to care coordination may be important for supporting continuity and coherence [11]. In complex care networks, community nurses may assess whether caregiver roles are clear and whether communication among family members and formal care providers is adequate. When coordination difficulties are identified, additional support for communication and shared care planning may be considered.

### Strengths and limitations

This study had several limitations. First, the cross-sectional design prevents causal inference and limits the ability to determine the temporal ordering between care network structure and depression. In addition, the observed association of the Public-centered and Mixed Care Networks with depression may partly reflect selection into these care arrangements; individuals with more complex care needs, limited family caregiving resources, or pre-existing depressive symptoms may have been more likely to rely on public services or on multiple caregivers. Longitudinal studies are needed to elucidate the directionality and mechanisms underlying these associations. Second, some structural characteristics of care networks, such as proximity and frequency of contact, were not included because of limitations resulting from the use of secondary data. Incorporating these network dimensions in future studies would allow for a more comprehensive understanding of care network dynamics. Third, contextual factors, such as relationship quality, caregiver burden, and stability of care arrangements, were not available in the dataset, limiting our ability to examine the pathways linking network structure to depression. Future studies should integrate these qualitative and contextual elements to better elucidate underlying mechanisms.

Despite these limitations, this study had several strengths. By employing latent class analysis, this study moves beyond traditional single-caregiver or predefined categorizations to empirically identify distinct care network types. This person-centered approach considers the combination of multiple caregivers within individuals, allowing us to capture complex, naturally occurring patterns that would not be detectable through simpler approaches. This provides a more nuanced, data-driven understanding of how care resources are organized for people with cognitive impairment. Additionally, this study fills an important gap by examining care networks in South Korea, a context in which family centered caregiving traditions differ substantially from those in Western societies. Finally, by incorporating an integrated theoretical framework and accounting for personal, health-related, and care adequacy factors, we identified how the care network structure could be associated with depression in people with cognitive impairment.

## Conclusion

Distinct care network types were identified among people with cognitive impairment, and the Public-centered and Mixed Care Networks were associated with higher depression scores compared with the Child-centered Care Network. These findings suggest that the structural composition of care networks is relevant to understanding depression in this population and highlight the importance of considering the overall configuration of caregivers, rather than focusing only on the primary caregiver. When developing care strategies for people with cognitive impairment, consideration should be given to the specific configuration of care networks, including the involvement of multiple caregivers and limited involvement of close informal caregivers. Psychological well-being should also be regularly monitored, particularly among those in Public-centered or Mixed Care Networks.

## References

[1] Leung DKY, Chan WC, Spector A, Wong GHY. Prevalence of depression, anxiety, and apathy symptoms across dementia stages: a systematic review and meta-analysis. Int J Geriatr Psychiatry. 2021;36(9):1330–44. doi: 10.1002/gps.5556 33905138

[2] Kim B, Noh GO, Kim K. Behavioural and psychological symptoms of dementia in patients with Alzheimer’s disease and family caregiver burden: a path analysis. BMC Geriatr. 2021;21(1):160. doi: 10.1186/s12877-021-02109-w 33663416 PMC7934246

[3] Mourao RJ, Mansur G, Malloy-Diniz LF, Castro Costa E, Diniz BS. Depressive symptoms increase the risk of progression to dementia in subjects with mild cognitive impairment: systematic review and meta-analysis. Int J Geriatr Psychiatry. 2016;31(8):905–11. doi: 10.1002/gps.4406 26680599

[4] Toot S, Swinson T, Devine M, Challis D, Orrell M. Causes of nursing home placement for older people with dementia: a systematic review and meta-analysis. Int Psychogeriatr. 2017;29(2):195–208. doi: 10.1017/S1041610216001654 27806743

[5] Hwang Y, Kim J. Influence of caregivers’ psychological well-being on the anxiety and depression of care recipients with dementia. Geriatr Nurs. 2024;55:44–51. doi: 10.1016/j.gerinurse.2023.10.015 37972435

[6] Kales HC, Gitlin LN, Lyketsos CG. Assessment and management of behavioral and psychological symptoms of dementia. BMJ. 2015;350:h369. doi: 10.1136/bmj.h369 25731881 PMC4707529

[7] Ning W, Wang S, Xu Y, Cheung D. The association between caregiver psychosocial factors and depressive symptoms in people with dementia: a systematic review and meta-analysis. J Adv Nurs. 2025;n/a. doi: 10.1111/jan.17093 PMC12994642 40556482

[8] McKhann G, Drachman D, Folstein M, Katzman R, Price D, Stadlan EM. Clinical diagnosis of Alzheimer’s disease: report of the NINCDS-ADRDA work group under the auspices of department of health and human services task force on Alzheimer’s disease. Neurology. 1984;34(7):939–44. doi: 10.1212/wnl.34.7.939 6610841

[9] 2024 Alzheimer’s disease facts and figures. Alzheimers Dement. 2024;20(5):3708–821. doi: 10.1002/alz.13809 38689398 PMC11095490

[10] Andersson MA, Monin JK. Informal care networks in the context of multimorbidity: size, composition, and associations with recipient psychological well-being. J Aging Health. 2018;30(4):641–64. doi: 10.1177/0898264316687623 28553797 PMC5786491

[11] Ellis KR, Koumoutzis A, Lewis JP, Lin Z, Zhou Y, Chopik WJ, et al. Conceptualizing and operationalizing collaboration among multiple caregivers of older adults. J Gerontol B Psychol Sci Soc Sci. 2023;78(Suppl 1):S27–37. doi: 10.1093/geronb/gbac139 36409283 PMC10010467

[12] Spillman BC, Freedman VA, Kasper JD, Wolff JL. Change over time in caregiving networks for older adults with and without dementia. J Gerontol B Psychol Sci Soc Sci. 2020;75(7):1563–72. doi: 10.1093/geronb/gbz065 31102533 PMC7424285

[13] Leggett AN, Haldar S, Tsuker S, Lai W, Nemmers N, Choi H, et al. Who’s on your team? Classifying dementia caregiving networks and associations with the well-being of caregivers and care recipients with dementia. J Gerontol B Psychol Sci Soc Sci. 2025;80(6):gbaf040. doi: 10.1093/geronb/gbaf040 39994746 PMC12059475

[14] Lin Z. Diversity and dynamics in care networks of older Americans. Socius. 2024;10:23780231231223906. doi: 10.1177/23780231231223906

[15] KEATING N, Otfinowski P, Wenger C, Fast J, Derksen L. Understanding the caring capacity of informal networks of frail seniors: a case for care networks. Ageing and Society. 2003;23(1):115–27. doi: 10.1017/s0144686x02008954

[16] Antonucci TC, Ajrouch KJ, Birditt KS. The convoy model: explaining social relations from a multidisciplinary perspective. Gerontologist. 2014;54(1):82–92. doi: 10.1093/geront/gnt118 24142914 PMC3894851

[17] van Groenou MB. Associations between care network types and psychological well-being among Dutch older adults. Int J Care Caring. 2020;4(2):215–33. doi: 10.1332/239788220x15833754379590

[18] Lee C-Y, Jeon Y-H, Fethney J, Watson K, Low L-F, Mowszowski L, et al. The association between caregiving context and the health and well-being of carers and their care recipients living with dementia: a cross-sectional study. J Adv Nurs. 2026;82(6):6154–67. doi: 10.1111/jan.70278 41074247

[19] Swinkels JC, Abbing J, Broese van Groenou MI. Why is the composition of older adults’ care network associated with psychological wellbeing: an application of the self-determination theory. Aging Ment Health. 2025;29(1):121–9. doi: 10.1080/13607863.2024.2373405 38958434

[20] Hu M, Freedman VA, Patterson SE, Lewis N. Shared care networks assisting older adults: New insights from the national health and aging trends study. The Gerontologist. 2023;63:840–50. doi: 10.1093/geront/gnac155 36190818 PMC10268586

[21] Jacobs MT, Broese van Groenou MI, Aartsen MJ, Deeg DJH. Diversity in older adults’ care networks: the added value of individual beliefs and social network proximity. J Gerontol B Psychol Sci Soc Sci. 2018;73(2):326–36. doi: 10.1093/geronb/gbw012 26912490

[22] Lai A. Impact of care-recipient relationship type on quality of life in community-dwelling older adults with dementia and their caregivers. 2024. https://macsphere.mcmaster.ca/handle/11375/29751 10.1177/08919887231215044 PMC11089829 37950653

[23] Kang E, Kim H, Jung C, Kim S, Lee SH, Joo B, et al. National survey of older Koreans. Seoul: Korea Institute for Health and Social Affairs. 2023. https://www.mohw.go.kr/board.es?mid=a10411010200&bid=0019&act=view&list_no=1483359&tag=&nPage=1

[24] Baek MJ, Kim K, Park YH, Kim S. The validity and reliability of the mini-mental state examination-2 for detecting mild cognitive impairment and Alzheimer’s disease in a korean population. PLoS One. 2016;11(9):e0163792. doi: 10.1371/journal.pone.0163792 27668883 PMC5036810

[25] Bae JN, Cho MJ. Development of the Korean version of the geriatric depression scale and its short form among elderly psychiatric patients. J Psychosom Res. 2004;57(3):297–305. doi: 10.1016/j.jpsychores.2004.01.004 15507257

[26] Lee SC, Kim WH, Chang SM, Kim BS, Lee DW, Bae JN. The use of the Korean version of short form geriatric depression scale (SGDS-K) in the community dwelling elderly in Korea. J Korean Geriatr Psychiatry. 2013;17:37–43.

[27] Lee HY, An JY, Jang SY. Factors influencing depressive symptom based on the type of care for older adults living at home. Korean Soc Nurs Res. 2024;8(2):1–12. doi: 10.34089/jknr.2024.8.2.1

[28] Zenebe Y, Akele B, W/Selassie M, Necho M. Prevalence and determinants of depression among old age: a systematic review and meta-analysis. Ann Gen Psychiatry. 2021;20(1):55. doi: 10.1186/s12991-021-00375-x 34922595 PMC8684627

[29] Sinha P, Calfee CS, Delucchi KL. Practitioner’s guide to latent class analysis: methodological considerations and common pitfalls. Crit Care Med. 2021;49(1):e63–79. doi: 10.1097/CCM.0000000000004710 33165028 PMC7746621

[30] Weller BE, Bowen NK, Faubert SJ. Latent class analysis: a guide to best practice. J Black Psychol. 2020;46(4):287–311. doi: 10.1177/0095798420930932

[31] Nylund KL, Asparouhov T, Muthén BO. Deciding on the number of classes in latent class analysis and growth mixture modeling: a monte carlo simulation study. Struct Equ Model A Multidiscip J. 2007;14(4):535–69. doi: 10.1080/10705510701575396

[32] Fox J, Monette G. Generalized collinearity diagnostics. J Am Stat Assoc. 1992;87(417):178–83. doi: 10.1080/01621459.1992.10475190

[33] MacKinnon JG, White H. Some heteroskedasticity-consistent covariance matrix estimators with improved finite sample properties. J Econometrics. 1985;29(3):305–25. doi: 10.1016/0304-4076(85)90158-7

[34] Lee YW. Policy issues and challenges in welfare policies for elderly care. Korea Development Institute; 2020.

[35] Lai W, Nemmers N, Tsuker S, Leggett AN. Exploring caregiving network characteristics for older adults living with cognitive impairment across race and ethnicity. Gerontologist. 2025;65(6):gnaf110. doi: 10.1093/geront/gnaf110 40096543 PMC12129109

[36] Wolff JL, Mulcahy J, Huang J, Roth DL, Covinsky K, Kasper JD. Family caregivers of older adults, 1999–2015: trends in characteristics, circumstances, and role-related appraisal. Gerontologist. 2018;58:1021–32. doi: 10.1093/geront/gnx093 28637266 PMC6215459

[37] Lambotte D, De Donder L, Van Regenmortel S, Fret B, Dury S, Smetcoren A-S, et al. Frailty differences in older adults’ use of informal and formal care. Arch Gerontol Geriatr. 2018;79:69–77. doi: 10.1016/j.archger.2018.05.018 30125830

[38] Cantor MH. Family and community: changing roles in an aging society. Gerontologist. 1991;31(3):337–46. doi: 10.1093/geront/31.3.337 1879709

[39] Chappell N, Blandford A. Informal and formal care: exploring the complementarity. Ageing and Society. 1991;11(3):299–317. doi: 10.1017/s0144686x00004189

[40] Bijnsdorp FM, Pasman HRW, Francke AL, Evans N, Peeters CFW, Broese van Groenou MI. Who provides care in the last year of life? A description of care networks of community-dwelling older adults in the Netherlands. BMC Palliat Care. 2019;18(1):41. doi: 10.1186/s12904-019-0425-6 31092227 PMC6521417

[41] Coe NB, Konetzka RT, Berkowitz M, Blecker E, Van Houtven CH. The effects of home care provider mix on the care recipient: an international, systematic review of articles from 2000 to 2020. Annu Rev Public Health. 2021;42:483–503. doi: 10.1146/annurev-publhealth-090419-102354 33395544 PMC8190630

[42] Köhler K, Dreyer J, Hochgraeber I, von Kutzleben M, Pinkert C, Roes M, et al. Towards a middle-range theory of “Stability of home-based care arrangements for people living with dementia” (SoCA-Dem): findings from a meta-study on mixed research. BMJ Open. 2021;11(4):e042515. doi: 10.1136/bmjopen-2020-042515 33853798 PMC8054086

[43] Penning MJ. Receipt of assistance by elderly people: hierarchical selection and task specificity. Gerontologist. 1990;30(2):220–7. doi: 10.1093/geront/30.2.220 2347504

[44] Song M-K, Paul S, Happ MB, Lea J, Pirkle JL Jr, Turberville-Trujillo L. Informal caregiving networks of older adults with dementia superimposed on multimorbidity: a social network analysis study. Innov Aging. 2023;7(4):igad033. doi: 10.1093/geroni/igad033 37197444 PMC10184695

